# A Comprehensive Study of the Impacts of Oat β-Glucan and Bacterial Curdlan on the Activity of Commercial Starter Culture in Yogurt

**DOI:** 10.3390/molecules25225411

**Published:** 2020-11-19

**Authors:** Marek Aljewicz, Małgorzata Majcher, Beata Nalepa

**Affiliations:** 1 Department of Dairy Science and Quality Management, Faculty of Food Science, University of Warmia and Mazury in Olsztyn, Oczapowskiego 7, 10-719 Olsztyn, Poland; 2 Faculty of Food Science and Nutrition, Poznań University of Life Sciences, Wojska Polskiego 31, 60-624 Poznań, Poland; malgorzata.majcher@up.poznan.pl; 3 Department of Industrial and Food Microbiology, Faculty of Food Science, University of Warmia and Mazury in Olsztyn, Pl. Cieszyński 1, 10-726 Olsztyn, Poland; bena@uwm.edu.pl

**Keywords:** volatile compounds, fermentation, polysaccharide, lactose, organic acid, bacterial β-glucan, viability, bacteria metabolism

## Abstract

This study provides important information about the impacts of various levels of oat (OBG) and bacterial (curdlan) β-glucan and fat contents in milk on survivability and metabolism of yogurt starter cultures. The results show that addition of β-glucans in the concentration higher than 0.25% reduced starter bacterial counts during storage and prolonged the milk acidification process. A significant increase in lactose consumption by starter cultures was noted in the yogurt samples with OBG addition up to 0.75%. The highest (by 567% on average) increase in lactic acid content was noted in the control yogurts. Whereas the lowest (by 351%) increase in lactic acid content was noted in yogurts with OBG. After 28-day storage, the acetic aldehyde content was significantly influenced by fat content, type and addition level of polysaccharide. A higher increase in acetoin content was noted in samples with 0.25% than in samples with 1% of polysaccharides. In turn, significantly lower increases in diacetyl and 2,3-pentanedione contents were observed in the yogurt samples with OBG than in these with curdlan, with diacetyl production increase along with the higher concentration of the polysaccharide. The addition of OBG and curdlan to milk contributed to differences in the starter culture metabolism, consequently, in the milk acidification dynamics.

## 1. Introduction

Functional foods include products, which, apart from providing specific nutrients to the body, exert a beneficial effect on consumer’s health. The best-known examples of functional foods are fermented milk and yogurts [1]. According to the Codex Alimentarius Standard [2], the minimum number of starter cultures in fermented milk beverages should not fall below log 7 cfu/mL or g throughout the storage period. Therefore, the addition of polysaccharides (commonly known as prebiotics) is used in their production process to maintain a certain number of bacterial cultures, stimulate growth, and increase the survival rate of probiotic cultures [3]. Yogurts are manufactured with various polysaccharides, the most common of which include: inulin, oligofructose, galactooligosaccharides, lactulose, starch, and guar gum [4,5,6]. Depending on the source of isolation, structure, or concentration, the polysaccharides serve different technological functions. They can be used as stabilizers, thickeners, or fat substitutes in low-calorie products (the so-called light type products).

One of the most promising polysaccharides featuring prebiotic properties is β-glucans (BGs). BGs are natural, non-ionic polysaccharides whose structure and properties depend on the isolation source [7]. For instance, β-glucan isolated from the *Agrobacterium* sp. bacteria (curdlan) has a linear structure in which the molecules of β-d-glucopyranose units are linked by a β-(1 → 3) bond [8]. In turn, β-glucans isolated from oats show a more complex structure, as they are linear homopolysaccharides composed of d-glucopyranosyl residues linked by a mixture of β-(1 → 3) and β-(1 → 4) bonds [9]. Besides imparting the appropriate functional features to food products, β-glucans may reduce the incidence of such diet-related diseases as hyperinsulinemia, hyperlipidemia, hypercholesterolemia, hypertension, or cancer, when consumed in appropriate amounts. It has been shown that the more complex the structure of β-glucan is, the greater its potential health-promoting effect [8,10]. Due to the rheological behavior that determines their technological and nutritional usefulness, BGs can be used to produce functional and nutraceutical foods [11].

Compared to inulin or carrageenan, BGs are characterized by high thermodynamic incompatibility with milk proteins. Depending on the addiction level and the source of isolation, they contribute to product delamination and whey separation during the milk acidification process. This process occurs faster when BG concentration and yogurt’s dry matter content are lower [9]. The thermodynamic changes taking place during milk acidification also significantly affect the metabolic activity of bacteria and the production of certain organic acids or volatile compounds typically found in yogurts [12]. The addition of prebiotics during yogurt production may disturb the bacterial metabolism, extend the fermentation time, or lead to changes in the amount and type of volatile compounds synthesized by bacteria. Few studies have shown that the use of exopolysaccharide-producing strains [13] or BGs in a concentration of up to 1% during sour milk or yogurt production led to a significant extension of the milk coagulation time [14]. Different results were achieved in the studies with 1–3% inulin addition. The dynamic of acidification was faster, along with its higher addition level [15]. The study by Vasiljevic et al. [16] showed that the addition of oat BG to yogurt resulted in improved survival of starter cultures and improved product storage stability. Similar results were also found in yogurt samples with 0.75% addition of barley BG and up to 0.5% addition of curdlan [10,17]. Despite the availability of several works [5,14,17], knowledge about the influence of different oat β-glucan concentrations on the fermentative transformations during yogurt production and storage is still skimpy. Furthermore, the comparison of the results reported by various authors is difficult due to preparations having different concentrations and added in different amounts. The scientific literature provides no information regarding the effect of fat content on fermentation dynamics, the survivability of starter cultures, and contents of volatile compounds in yogurts produced with various inclusion levels of BG isolated from bacteria (curdlan).

Therefore, this study was undertaken to enable a comprehensive, comparative assessment of the effect of different addition levels of two highly purified β-glucan preparations isolated from oats and bacteria to milk with different fat contents on the dynamics of milk acidification, the survival of starter cultures, sugar metabolism, and the synthesis of organic acids and volatile compounds typical of yogurts.

## 2. Results and Discussion

### 2.1. Effect of the Addition Level of Glucan Preparations on Changes in the Counts of Starter Cultures

**Figure 1:**
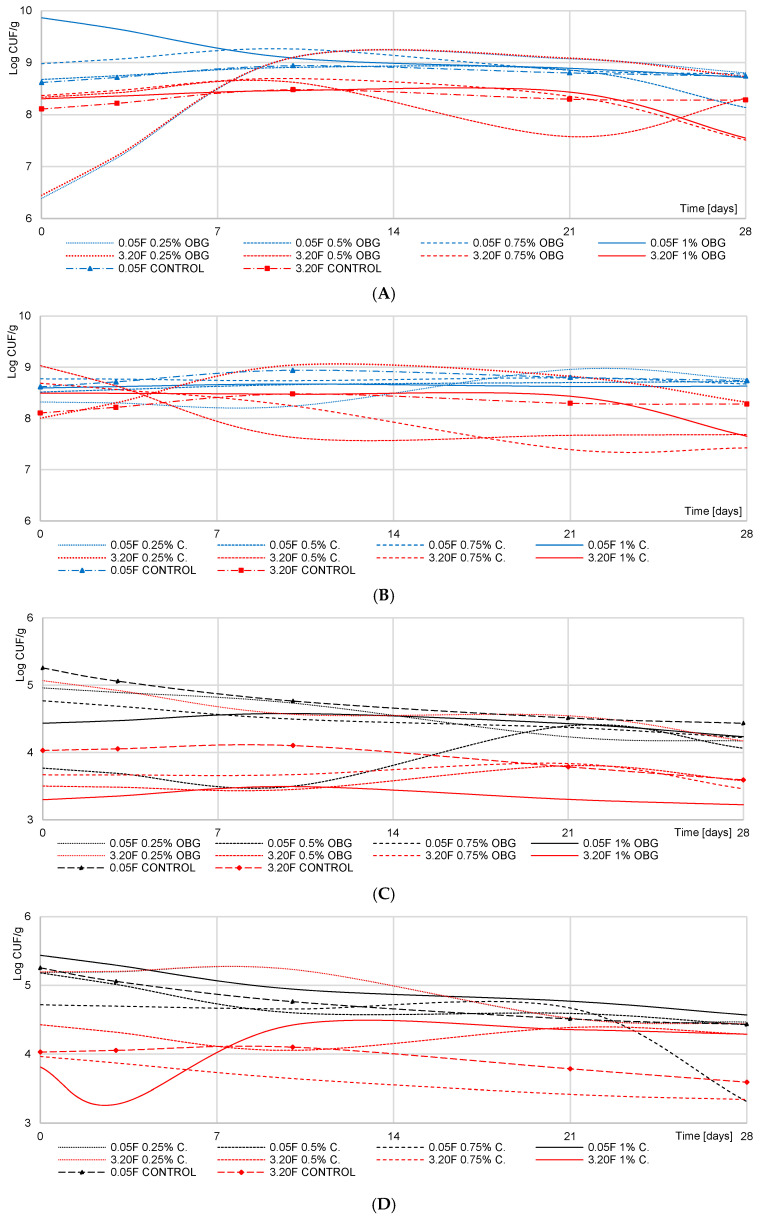
Viability of starter lactic acid bacteria over 28 days of storage of yogurt. (**A**,**B**)—*Streptococcus thermophiles* count; (**C**,**D**)—*Lactobacillus dulbrecki* ssp. *bulgaricus* count; OBG—yogurt with oat β-glucan; (**C**)—yogurt with bacterial β-glucan (curdlan); F-fat content. The 0 is the time when the milk reaches pH 4.6 and starts refrigerated storage.

The mean *Streptococcus thermophilus* (ST) count in the control yogurts produced from skimmed milk and full-fat milk reached 8.73 log cfu/g (Figure 1; Table S1). The addition of OBG and curdlan led to, respectively, significantly higher (ca. 0.5 log cfu/g) and lower (ca. 0.4 log cfu/g) ST counts after completed fermentation, compared to the control yogurts. In turn, no significant differences in ST numbers were noted between experimental and control yogurts in the study with a structurally-similar polysaccharide-pullulan [18] because of carbon sources’ presence in milk that was easier available to bacteria than pullulan.

After 28 days of storage, regardless of fat content in the raw material, significant changes were noted in the ST population in all yogurts analyzed. A significant (*p* < 0.001) increase in the ST count was determined only in the yogurts with a 0.25% addition of OBG and curdlan, reaching 50% (~3 log cfu/g) and 7% (~0.6 log cfu/g) in these made of skimmed milk as well as 17% (~1.3 log cfu/g) and 5% (~3 log cfu/g) in these made of full-fat milk, respectively. These observations are consistent with findings reported by Kilic et al. [5], who showed that a 0.25% addition of a PROMOAT preparation (with 29% content of β-glucan) increased ST population. When used in higher than 0.25% concentrations, both preparations decreased ST count, which is in line with Lazaridou et al. [9], and Zhao et al. [10]. In contrast to our study, the use of a 2% addition of pullulan [18] or inulin [19] caused no significant change in ST count compared to the control yogurt, which indicates a significant effect of preparation structure on ST metabolism.

The mean count of *Lactobacillus bulgaricus* (LB) in yogurts was lower than that of ST and reached 5.19 log cfu/g in the yogurts made of skimmed milk and 4.03 log cfu/g in these made of full-fat milk (Figure 1). These values are ca. 30% lower than data presented by Kycia et al. [18], which is due to the use of starter cultures with different ST to LB ratios and at different addition levels. The results of the multi-way analysis of variance showed that LB count determined after 28 days of storage was significantly affected by polysaccharide type and addition level (*p* < 0.01) (Table S7), which is consistent with the findings presented by Lazaridou et al. [9]. After 28-day storage of yogurts made of full-fat milk, the population number of LB increased by 7.30% in the samples with OBG addition and by 2.25% in these with curdlan addition. In turn, the LB count decreased slightly (*p* > 0.05) in the yogurts made of skimmed milk, probably because of the smaller availability of carbon and nitrogen sources to the bacteria, compared to the full-fat milk. These results are in line with data reported by Kycia et al. [18] for pullulan and with these presented by Kilic et al. [14] for OBG, whose addition to milk reduced *L. plantarum* count. An insignificant increase in the LB population was also demonstrated in yogurts with inulin addition [16]. Vasiljevic et al. [16] also concluded that OBG addition to yogurts produced from skimmed milk caused no significant LB count changes than the control yogurts, which agrees with the results of our study. In turn, Allgeyer et al. [4] demonstrated ca. 2 log cfu/g lower viability of a probiotic *Lactobacillus acidophilus* LA-5 strain after 30-day storage of yogurt drinks with maize fiber, polydextrose, and inulin. Unlike plant-based beverages, milk is a good source of carbon and nitrogen for starter cultures used to produce fermented milk beverages. According to some authors like Kilik et al. [5], lactic acid bacteria (LAB) count increases in yogurts with polysaccharides because they synthesize enzymes enabling the degradation of β-glucans to easily-available carbon sources. However, lactose or glucose represents a carbon source more easily available to bacteria than the polysaccharide added.

### 2.2. Effect of β-Glucan Addition on Milk Acidification Dynamics

**Figure 2:**
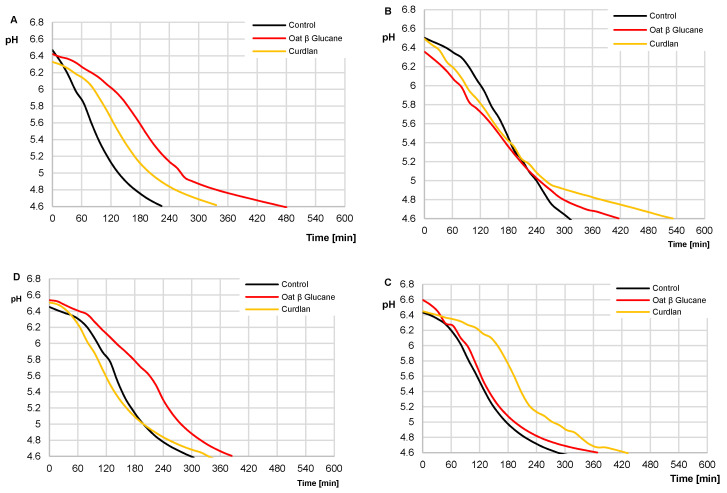
Dynamics of milk acidification depending on the addition of oat and bacterial β-glucan (curdlan). (**A**)—0.25% content of β-glucan; (**B**)—0.50% content of β-glucan; (**C**)—0.75% and (**D**)—1% content of β-glucan.

The pH value of control milk, milk with OBG, and milk with curdlan measured immediately after starter culture addition reached 6.46, 6.48, and 6.44, respectively, and was typical of the pH value of fresh milk after pasteurization (Figure 2).

The use of OBG and curdlan in the concentration of 0.25% significantly elongated milk acidification time, i.e., the time needed for milk acidification to pH = 4.6 ± 0.01 (compared to the control yogurt), to 256 min and 112 min (Figure 2A). These results are in line with the study by Kilic et al. [14], in which the use of BG preparations in the concentrations from 0.25% to 1% slowed down yogurt acidification dynamics. Similar observations were made by Kristo et al. [13], who demonstrated that the time needed for milk acidification to pH 4.6 was significantly longer upon using exopolysaccharide-producing strains than traditional starter cultures.

Opposite results were achieved by Lazaridou et al. [9], who used 1.4% OBG addition and did not observe any significant changes in milk acidification dynamics. It needs to be emphasized that OBG concentrations used in the present study were lower than those used by Lazaridou et al. [9]. Besides, in the present study, the milk acidification time shortened significantly, along with the OBG content increase in the sample (Figure 2B–D). Presumably, increasing its content to ca. 1.4% would cause similar results as those reported by Lazaridou et al. [9]. Our study’s results confirm that the metabolic activity of *Streptococcus thermophilus* can be significantly affected by a gel’s rheological properties. Probably, the gel’s high viscosity impaired the diffusion of essential growth substrates and the removal of bacterial metabolites.

### 2.3. Effect of β-Glucan Addition on Sugar Metabolism

**Figure 3:**
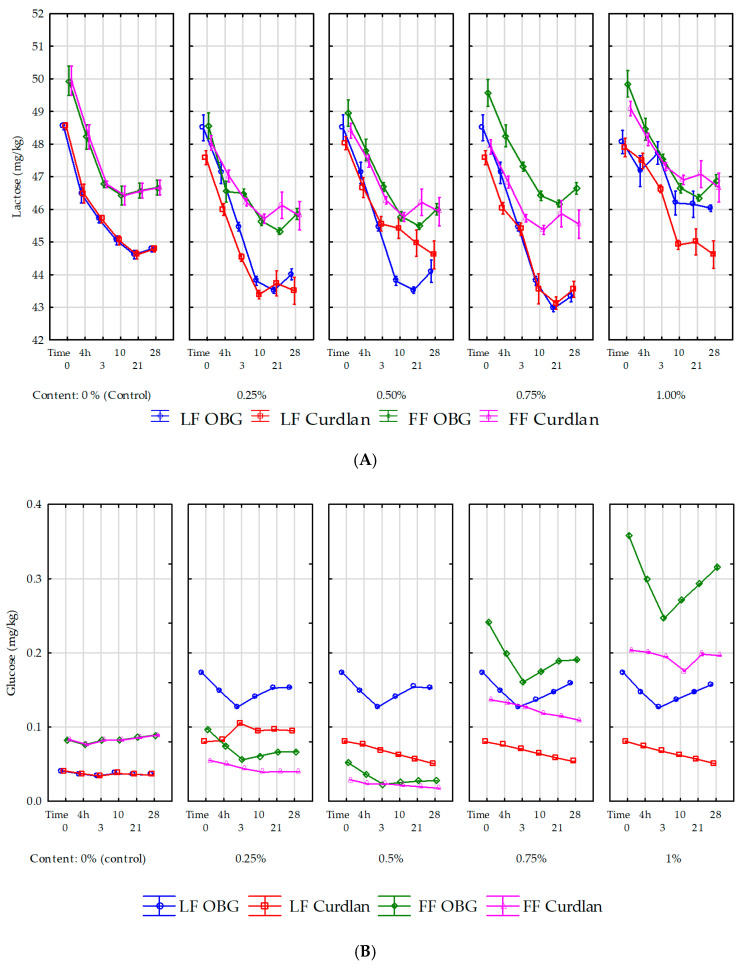
Lactose (**A**) and glucose (**B**) in yogurt samples with 0%, 0.25%, 0.5%, 0.75% and 1% content of oat β-glucan (OBG) and bacterial β-glucan (curdlan) overproduction and 28 days of storage. LF–Low fat (yogurt with 0.05% milkfat); FF–Full fat (yogurt with 3.2% milkfat). The values represent the mean and bars are represented the standard deviation. On the timeline, the time from 0 to 4 h is the milk acidification time; the value of 4 h is the time when the milk reaches pH 4.6 (yogurt production is finished) and starts refrigerated storage.

The changes in numbers of starter cultures over yogurt fermentation and storage periods are determined by the content and availability of easily-available carbon sources, i.e., carbohydrates. The mean lactose content in all experimental samples reached 48.70 g/L (Figure 3A, Tables S2 and S3).

The results are similar to those reported by Leite et al. [20], but lower than those presented by Delgado-Fernández et al. [21] for samples of yogurt made of cow’s milk with the addition of oligosaccharides (53–54 g/L). A lower mean lactose content was probably attributable to the milk chemical composition’s seasonal variations and a different normalization of the milk. Among all yogurt samples tested, the greatest (*p* < 0.05) decrease in lactose content was determined in the samples between day 0 and day 10 of storage. Since day 10 of storage, changes in lactose content were small and statistically insignificant. Lactose consumption by LAB was higher by ca. 5% in the experimental yogurts with 0.5% and 0.75% OBG, compared to the control yogurt and yogurt with curdlan (Table S6). In turn, in the samples with 1% addition of the polysaccharides, lactose consumption by LAB was comparable with that noted in the control samples (*p* > 0.05). Unlike curdlan, OBG probably stimulates bacteria’s metabolic activity; hence lower lactose contents were determined in the experimental yogurts.

Our study results are consistent with those reported by Pereira da Costa et al. [12], who noted a significant decrease in lactose content in yogurt samples with a 10% addition of cupuassu pulp. The above authors and also Zalán et al. [22] demonstrated that the use of inulin in the production of yogurt does not significantly increase lactose metabolism, which could be due to use of the prebiotic as a carbon source by starter lactic acid bacteria (SLAB). Nevertheless, given that milk also contains glucose, the polysaccharide utilization as the first carbon source of choice seems disputable. The glucose is consumed by SLAB in metabolic processes. Our study showed its content significantly (*p* < 0.01) depended on storage time and product type. Regardless of sampling time, the highest glucose content was determined in the yogurts with OBG and the lowest in the control products (Figure 3B). In the yogurts with OBG, glucose content decreased significantly until day 3 of storage and then increased slowly. This dependency was, probably, due to an insignificantly higher LAB count in the yogurts with OBG at the early stage of storage (Figure 1). However, the significant increase observed since day 3 of storage was most likely because the bacteria found another easily available carbon source. Compared to the yogurt with OBG, a successive, significant decrease in glucose content was noted in the yogurt with curdlan. In turn, in the control yogurts, statistically insignificant changes in its content were observed throughout the storage period. These results agree with the study by Pereira da Costa et al. [12]. Changes in glucose content in yogurts during their storage are strongly associated with lactose metabolism. Glucose synthesized in the Embden-Meyerhoff-Parnas pathway is hydrolyzed by bacteria firstly to pyruvate and then to lactate. Therefore, changes in its content in a product may be one indicator of polysaccharide’s effect on bacteria’s metabolic activity.

### 2.4. Effect of β-Glucan Addition on Changes in the Contents of Organic Acids in Yogurt

**Figure 4:**
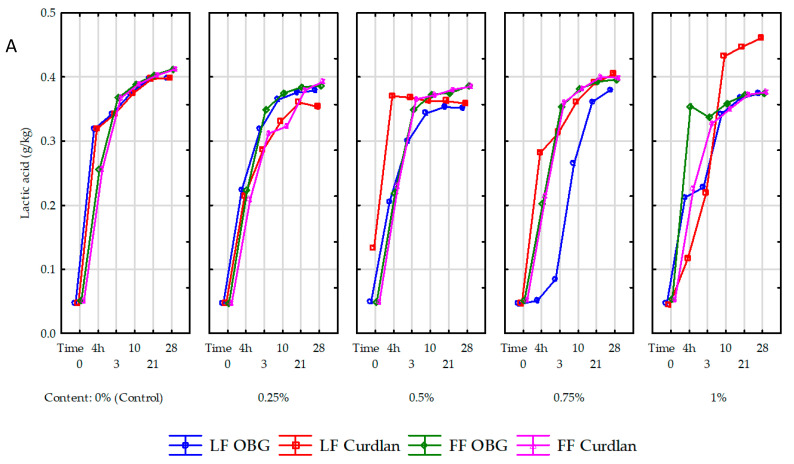

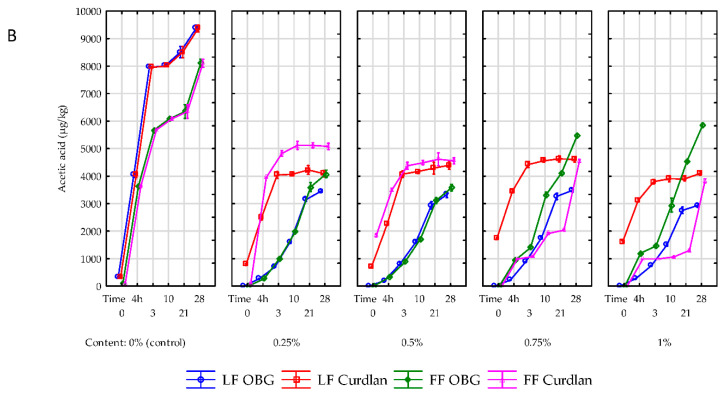
Lactic (**A**) and acetic acid (**B**) in yogurt samples with 0%, 0.25%, 0.5%, 0.75% and 1% content of oat β-glucan (OBG) and bacterial β-glucan (curdlan) overproduction and 28 days of storage. LF–Low fat (yogurt with 0.05% milkfat); FF–Full fat (yogurt with 3.2% milkfat). The values represent the mean and the bars are represented the standard deviation. On the timeline, the time from 0 to 4 h is the milk acidification time; the value of 4 h is the time when the milk reaches pH 4.6 (yogurt production is finished) and starts refrigerated storage.

Specific metabolic pathways of bacteria lead to the conversion of sugars into organic acids and volatile compounds. Depending on the strain and species composition of starter cultures, lactose fermentation may proceed via the glycolytic pathway, during which only lactic acid is produced, or via the phosphate-pentose pathway ending in the synthesis of not only lactic acid but also acetic acid [23]. Lactic acid turned out to be the major acid among all organic acids determined (Figure 4, Tables S4 and S5).

The lactic acid content in the samples before the acidification process depends on the microbial quality of raw milk used to produce. In the present study, its content of lactic acid in milk before inoculation with the starter culture ranged from 0.045–0.053 g/L (Figure 4A). A significant (*p* < 0.001) increase in lactic acid content was determined in yogurt samples during the acidification process, due to the proliferation of starter cultures and lactose metabolic conversion by starter cultures to glucose and then, i.e., to lactic acid. The amount of organic acids synthesized during fermentation depends not only on the starter culture type but may be either increased or decreased by the functional additives used [24], including OBG or curdlan.

This was also confirmed in our study, where the highest (0.271 g/L; 567% on average) increase in lactic acid content during fermentation was noted in the control yogurts and the lowest one (0.165 g/L; 351% on average) in the yogurts with OBG. Our results are similar to the results reported by Kilic et al. [14], who showed the 0.25–1% BG addition to milk suppressed synthesis of lactic acid by starter cultures. Compared to the yogurt with OBG, the lactic acid content in the yogurts with curdlan was significantly dependent on polysaccharide addition level. The highest (0.370 g/L) lactic acid content was determined in the sample with 0.5% addition and the lowest one (0.117 g/L) in that with a 1% addition of curdlan. Worthy of attention is that over the ripening process, the increase in lactic acid content was significantly higher in the yogurt samples made of full-fat milk with OBG (0.200 g/L; 395%) than in the yogurts made of skimmed milk (0.165 g/L; 351%). Presumably, the coupling of fat globule elements released during yogurt production with OBG enhanced bacterial cultures’ metabolic activity, consistent with the study by Moe et al. [25]. An increased mean lactic acid content, reaching 0.398, 0.371, and 0.394 g/L in the control yogurts and these with OBG and curdlan, respectively, and significantly (*p* < 0.01) influenced by polysaccharide preparation type and addition level was also observed after 28-day storage. Interestingly, in the yogurts made of skimmed milk with curdlan addition, its content increased along with the polysaccharide inclusion level. The results obtained over the fermentation period and after 28-day storage point to a significant effect of the polysaccharide itself (its structure) and the concentration of a polysaccharide preparation on the course of glycolytic transformations mediated by SLAB. The above results are in line with works by Özer et al. [6] and Li et al. [26], who demonstrated the use of such polysaccharides as lactulose, inulin, iso-maltooligosaccharides, and xylooligosaccharides led to a significant (ca. 2 g/kg) increase in the lactic acid content of the experimental yogurts. As in the case of lactic acid, the content of acetic acid was also significantly affected by polysaccharide addition level, storage time, and fat content in the raw material used to produce yogurts. After completed fermentation of yogurts made of skimmed milk, the highest increase in acetic acid content (1169%; 3699 µg/kg) was determined in the control samples (Figure 4B); while in the yogurts with OBG and curdlan this increase was significantly (*p* < 0.01) lower and reached 100% (244 µg/kg) and 250% (1622 µg/kg) on average, respectively. In addition, acetic acid content was higher in the yogurts made of full-fat than skimmed milk, which contradicts the results reported by Kaminarides et al. [27]. Compared to the fermented yogurt samples, in the same samples stored for 28 days, the smallest (156%; 1462 µg/kg) increased in acetic acid content was determined in the control samples, whereas the largest one (1380%; 3059 µg/kg) in the yogurt with OBG. The increased content of acetic acid in yogurts was due to various metabolic transformations of LAB involved in, i.a., fermentation of lactose and degradation of citrates or amino acids [24]. The amount of synthesized acid was also significantly (*p* < 0.01) dependent on the polysaccharide addition level. The polysaccharide addition of up to 0.75% led to an increase, whereas its higher addition at 1% caused a decrease in acetic acid content in the yogurts, which is partly in line with results obtained by Kilic et al. [5], who demonstrated that acetic acid content was proportional to the inclusion level of an OBG preparation (PROMAT). However, in contrast to our study, the authors cited above used a preparation in which OBG accounted for only 30%. The higher acetic acid growth dynamics in the yogurt samples with OBG than curdlan agrees with other authors’ observations. The use of only apparently similar preparations of BG isolated from cereals, i.e., oats or barley, increased acetic acid content in the yogurt samples with barley β-glucan compared to these with oat β-glucan [24]. In turn, the use of iso-maltooligosaccharides and xylooligosaccharides caused no significant changes in its content in yogurt samples [26], whereas results of studies with inulin were inconclusive [6,26], probably due to various inulin preparations and starter cultures used.

### 2.5. Effect of β-Glucan Addition on the Changes in Contents of Volatile Compounds

**Figure 5:**
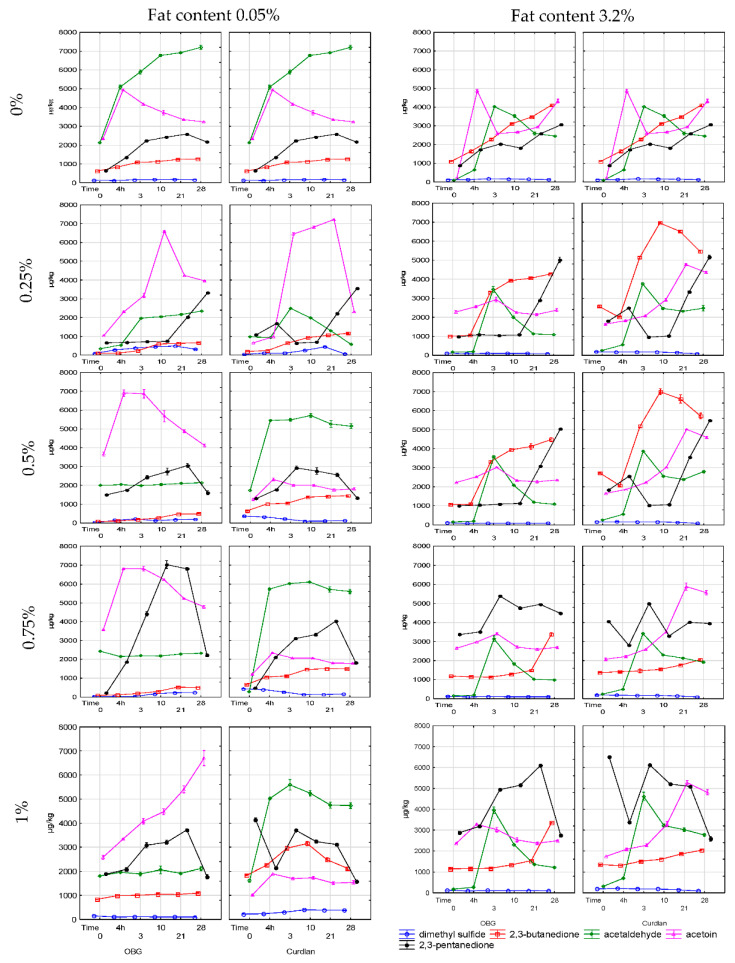
Profile of volatile compounds in yogurt with 0.25%, 0.5%, 0.75% and 1% content of oat and bacterial (curdlan) β-glucan during production and over 28 days of storage at 4 °C. Values are means ± standard deviation. On the timeline, the time from 0 to 4 h is the milk acidification time; the value of 4 h is the time when the milk reaches pH 4.6 (yogurt production is finished) and starts refrigerated storage.

Contents of volatile compounds, i.e., dimethyl sulfide, acetaldehyde, diacetyl, acetoin, and 2,3-pentanedione, in yogurts, are presented in Figure 5. Together with organic acids and sugars, volatiles of yogurts determine their sensory quality. Dimethyl sulfide content in the yogurts made of skimmed and full-fat milk with the addition of polysaccharides was at 127 µg/kg and 116 µg/kg, and accounted for ca. 1.5% of total volatile compounds determined (Figure 5).

After completed fermentation, a significant increase in dimethyl sulfide content was only observed in the yogurts with OBG. In the samples with curdlan, its content decreased depending on the polysaccharide addition level (Figure 5, Table S7). These results are similar to the findings presented by Ott et al. [28] and significantly lower than those from the study by Martin et al. [29], where dimethyl sulfide content in yogurts was as high as 10,000 µg/kg. This difference was attributable to a different yogurt production technique and analytical method used for dimethyl sulfide determination.

As in the fermentation case, a significant (*p* < 0.01) increase was noted in dimethyl sulfide content during storage of yogurts with OBG addition of up to 0.75%, with the increase being proportional to OBG addition level. Besides, a more significant increase in dimethyl sulfide content was determined in the yogurts made of skimmed than full-fat milk. In turn, in most yogurts with curdlan, dimethyl sulfide’s content decreased significantly (~50%), proportionally to its addition level. These results are in line with those reported by Moineau-Jean et al. [30], who also demonstrated that dimethyl sulfide content decreased by ca. 50% over a 28-day storage period.

The changes observed in dimethyl sulfide content over the storage period and its various contents in the yogurts examined are due to the various effects of the β-glucan preparations on the metabolic activity (conversion of amino acids) and, to a lesser extent, the count of *L. dulbrecki* ssp. *bulgaricus*. However, considering the study results, determining the effect of polysaccharides on dimethyl sulfide content is not utterly possible and requires further research.

Acetaldehyde is one of the key compounds determining the typical, fresh aroma of yogurts. It is produced by *Lactobacillus* via more than one metabolic pathway, from various precursors, including lactose, valine, pyruvate, acetyl phosphate, and—most of all—threonine [27,28]. In the control samples made of skimmed milk, its mean content reached 2118 µg/kg and was significantly (*p* < 0.001) higher than in the yogurts made of full-fat milk (83 µg/kg) (Figure 5).

The high acetaldehyde content in milk before inoculation is probably, due to the microbiological quality of raw milk. After completed fermentation, significantly higher acetaldehyde contents were determined in the yogurts produced from skimmed milk and full-fat milk, i.e., 5084 µg/kg and 633 µg/kg, respectively. The acetaldehyde contents determined in these yogurts were significantly higher than those presented by Kaminarides et al. [27] (180 µg/kg). The addition of the polysaccharide preparations to yogurts led to acetaldehyde content decrease, consistent with the study by Elsanhoty et al. [17], who used 0.75% addition of OBG of unknown purity. In addition, acetaldehyde production by bacteria was significantly dependent on polysaccharide’s structure; namely, the highest increase (242%; 3145 µg/kg) in its content was noted in the yogurts with curdlan featuring a linear structure and the smallest one (122%; 126 µg/kg) in the yogurt with OBG which structure is a more complex. Also, a greater increase in acetaldehyde content was shown in the yogurts made of skimmed than full-fat milk.

After 28-day storage of yogurts, the content of acetaldehyde depended significantly (*p* < 0.001) on fat concentration in milk as well as preparation type and additional level. The yogurts with OBG were greater (528%; 879 µg/kg) increase was determined in the yogurts made of full-fat than skimmed milk (188%; 558 µg/kg). In turn, in the yogurt samples with curdlan, the acetaldehyde content decreased in these made of skimmed milk (−13% on average; 276 µg/kg), but increased by 435% (1912 µg/kg) in these made of full-fat milk, which is associated with oxidative changes taking place in yogurts during storage [31]. The presented results stay in opposition to the study by Elsanhoty et al. [17], who demonstrated that a 0.75% addition of an OBG preparation (with unknown β-glucan concentration) led to acetaldehyde content decrease. Considering the yogurt cultures used (*L. dulbrecki* ssp. *bulgaricus* and *Str. termophilus*), and the fact that traditional SLAB cultures do not synthesize enzymes that metabolize acetaldehyde to, i.a., ethanol, acetaldehyde content should rather increase than decrease. Nevertheless, genetically-modified strains can decrease acetaldehyde content and increase ethanol content in yogurt samples [32]. Besides, the higher acetaldehyde content in the yogurts with OBG was, probably, due to their manufacture method (longer time of mixing the raw material and higher temperature of glucan preparation dissolution), resulting in better milk aeration before the inoculation, which enhanced aldehyde synthesis by SLAB [27,31].

2,3-Pentanedione and diacetyl represent ketones produced in yogurts via β-oxidation of saturated fatty acids and decarboxylation of β-keto acids. Diacetyl accumulated in yogurt is then converted to acetoin by diacetyl reductase [32]. After completed fermentation, a significantly higher increase in diacetyl content was noted in the yogurts with OBG than in these with curdlan, with the amount of diacetyl produced being higher (*p* < 0.001), the higher was the polysaccharide concentration (Figure 5). A similar dependency was observed for 2,3-pentanedione. After 28-day storage, diacetyl content increased significantly in the yogurt samples. The highest increase in its content was determined in the yogurts made of skimmed and full-fat milk with OBG and reached, on average, 420% (358 µg/kg) and 353% (2768 µg/kg), respectively. In the respective yogurt samples with curdlan addition, the increase in its content was smaller and reached, on average, 216% (415 µg/kg) and 213% (2117 µg/kg), respectively. Regardless of the preparation used, the highest increase in diacetyl content was noted in the yogurts with 0.25% addition and the lowest one—in these with 1% addition of the polysaccharide, which indicates a significant effect of polysaccharide concentration on the potential consumption of, e.g., glucose, during diketone synthesis [33]. The above results are consistent with those presented by Moineau-Jean et al. [30], who showed a significant increase (ca. 300%) in diacetyl content over 38-day storage. In another research, during 28-day storage, diacetyl content decreased significantly in control yogurts and yogurts with 0.75% addition of OBG with unknown concentration [17]. In contrast to the above-cited authors’ findings, our study results are due to different starter cultures used. Besides, as in the case of acetaldehyde, a different yogurt production method (longer heat-treatment, intensive aeration) enhanced oxidative transformations and increased diacetyl content in the samples with OBG. Worthy of notice is also the higher diacetyl content in the samples with 0.25% than 1% addition of the polysaccharide. Due to the high incompatibility of OBG and milk proteins, lower concentrations of the polysaccharide led to phase separation in the yogurts. Presumably, diacetyl synthesis proceeded mainly in whey, where the osmotic pressure is lower and, by this means, SLAB’s metabolic activity is higher. Study results also confirm that the raw material was an important source of diacetyl (buttery aroma) and that the fermentation process had a negligible effect on diacetyl content increase, which is consistent with the observations made by Routray and Mishry [34] and confirms that certain aromas are developed in yogurts in other transformations not necessarily associated with lactic fermentation.

The metabolic activity of bacteria and biochemical transformations taking place during yogurt production or storage results in an increased acetoin content [32]. The statistical analysis demonstrated that the acetoin content of yogurt samples was significantly affected by product type, storage time, polysaccharide addition level, and fat content in the raw material (*p* < 0.01) (Table S7). After completed fermentation, the average increase in acetoin content in the control yogurt reached 215% (2627 µg/kg), whereas, in the experimental yogurt, it was smaller and reached 147% on average. The acetoin content increase was higher in the yogurts made of skimmed than full-fat milk. Furthermore, it was higher in the yogurts with 0.25% than 1% addition of the polysaccharide, which was in line with the study concerning inulin addition [35]. After 28-day storage, acetoin content decreased in the control samples, with a greater decrease observed in the yogurts made of skimmed milk (−56%; 1694 µg/kg) than full-fat milk (−11%; 538 µg/kg). A decreased acetoin content was determined in the yogurts made of full-fat milk with OBG addition and in these made of skimmed milk with curdlan addition; with the decrease being the greater, the higher was polysaccharide addition level. Most likely, in these products, acetoin was transformed into 2,3-butanediol by acetoin reductase. In turn, acetoin and diacetyl contents were observed to increase in the yogurt samples made of full-fat milk with curdlan addition. Probably, curdlan addition contributed to the modification in LAB metabolism due to which the higher acetoin content in the yogurts was mostly attributed to the activation of biochemical pathways enabling its synthesis from alfa-acetolactate [32]. Our study results are consistent with works [14], which demonstrated that diacetyl content depended on the starter culture’s polysaccharide and strain composition [14]. Those authors also showed more enhanced diacetyl synthesis in the yogurt samples with inulin than in these with OBG. Changes in the concentrations of volatile compounds and other aroma-related compounds during storage are mainly due to the reactions in which they are synthesized or transformed into other compounds by bacterial metabolic enzymes and their losses caused by their oxidation [36]. The literature provides incomplete data on the effect of OBG and—most of all—curdlan on the contents of various aroma compounds in yogurts. However, some studies have shown that OBG can adversely affect the content of volatiles in cheeses [37].

## 3. Materials and Methods

### 3.1. Materials

The study was performed on milk with 0.05% and 3.2% fat content. The experimental yogurts were produced with the addition of highly-purified (75%; Mw = 0.97 × 10^6^ g) β-glucan isolated from oats *Avena sativa* L. (Beta Bio Technology, Częstochowa, Poland) and highly purified (90%; Mw = 0.91 × 10^6^ g) curdlan isolated from *Agrobacterium* sp. bacteria (Xi’an Lyphar Biotech Co., Ltd., Xian, China). The addition of oat β-glucan was normalized to ensure that β-glucan content was identical to that of the experimental yogurts containing curdlan. [^2^H_6_]Dimethyl sulfide, [^13^C_4_]2,3-butanedione, [^2^H_4_] acetaldehyde, d-glucose, d-galactose, lactose monohydrate, hexane, and H_2_SO_4_ were purchased from Sigma-Aldrich (St. Louis, MO, USA).

### 3.2. Organisation of the Experiment

In this article, two types of data are presented. The first type of data presents yogurt production time; in this period, time 0 is taken as the time before milk inoculated with starter cultures, “4 h” presents data for milk acidified to pH 4.6 (yogurt). The second type of data describes the storage time of the yogurt. During this period, the value “4 h” means that the yogurt is transferred to the cooling chamber and the storage period begins.

### 3.3. Yogurt Production

Yogurts were produced under laboratory conditions according to the patent application PL235801, by acidifying milk with starter cultures FD-DVS YC-X11 Yo-Flex (Chr. Hansen, Hørsholm, Poland). The experimental yogurt also contained 0.25; 0.5; 0.75 or 1% *w/w* of oat β-glucan or curdlan.

### 3.4. Microbiological Analysis

Microbiological examinations were carried out according to the PN-EN ISO 7218:2008 standard [38]. The total counts of *Streptococcus thermophilus* were determined on M17 agar (Merck, Darmstadt, Germany) in the samples incubated aerobically at 45 °C for 48 h (thermophilic cultures). Total *Lactobacillus dulbrecki* ssp. *bulgaricus* counts were determined on Rogosa agar (Merck) in the samples incubated anaerobically at 37 °C for 72 h in the AnaeroGen system (Oxoid, Poznan, Poland).

### 3.5. The Acidification Activity

The acidification activity was measured using a microelectrode and the Cerko Lab System (Cerko Lab, Gdynia, Poland). The pH was read at 1-min intervals. The measurement was performed at 43 °C. Reading was taken until the sample reached pH = 4.6.

### 3.6. Isolation of Volatiles

Volatiles were extracted by solid-phase microextraction with a carboxene/PDMS fiber (Supelco, St. Louis, MO, USA). For each analysis, 14 g of a sample was placed in 20-cm^3^ vials and spiked with a mixture of internal standards: [^2^H_6_] dimethyl sulfide, [^13^C_4_] 2,3-butanedione, and [^2^H_4_] acetaldehyde to reach 300 ppb concentration and sealed with a magnetic cap provided with a PTFE/silicone septum. The CTC Combipal autosampler (Agilent Technologies, Santa Clara, CA, USA) was used to extract volatiles. The sample vial was heated up to 50 °C for 5 min to equilibrate, and then the septum was pierced with an SPME needle. The fiber was exposed to a headspace of the sample for 45 min and after extraction was desorbed at 260 °C for 5 min.

During the injection, the fiber was exposed for 5 min in the splitless mode (1 min purge time). The chemical compounds were identified using a multidimensional gas chromatograph coupled to a time-of-flight mass spectrometer (GCxGC-ToF-MS, Pegasus IV, LECO; St. Joseph, MI, USA). The GC was equipped with an SPB-5 column (30 m × 0.32 mm × 0.25 μm; Supelco) and Supelcowax 10 (1 m × 0.1 mm × 0.1 μm; Supelco) as the second column. The operating GC conditions were as follows: helium flow rate 0.8 mL s^−1^, injection temperature 220 °C. For two-dimensional analysis, modulation time was optimized and set at 3 s, and mass spectra were collected at the electron ionization mode with ion source temperature at 220 °C, mass range *m/z* 33–330, acquisition rate of 150 scans s^−1^, and detector voltage 1750 V. Volatiles were identified by a comparison of their mass spectra and retention indices (RI) with data from The National Institute of Standards and Technology (NIST, Gaithersburg, MD, USA) library and respective standards. A mixture of *n*-alkanes (C_7_-C_20_) dissolved in hexane for retention index determination. The calculation was done using Chroma TOF software (version 4.50). The concentrations of volatiles in the sample were calculated from the analyte’s peak area and its corresponding internal labeled standard obtained for selected ions given in Table 1.

### 3.7. Lactic Acid Analysis

Lactic acid was extracted using a modified method described by Ferreira Barros et al. [39]. Briefly, 5 mL of 0.05 M H_2_SO_4_ was added to 1 mL of the sample and homogenized by vortexing for one minute. Afterward, the sample remained under agitation for one hour in an oven at 40 °C, and then it was centrifuged at 5000× *g* for 30 min at 4 °C. Finally, the supernatant was filtered through the 0.45 µm RC disposable syringe membrane filter (Sigma-Aldrich). The filtered samples were injected (20 µL) in triplicate into an Agilent 1260 (Agilent Technologies) HPLC system with DAD detector (at 215 nm) for lactic acid analysis. The determination of lactic acid was carried out using Supelcosil 610H, 300 × 7.8 mm cation-exchange column equipped with a cation H+ microguard cartridge (Supelco), maintained at 60 °C. The mobile phase used was 0.05M H_2_SO_4_, an isocratic flow rate of 0.5 mL min^−1^. Lactic acid was identified according to their retention times compared with standard solutions. The analysis was externally calibrated using mixed standard solutions in Milli-Q^®^ water (Millipore, Billerica, MA, USA), prepared for the samples. The concentration of each sample component was determined from the area of individual peaks. The standard curve coefficient was 0.9991.

### 3.8. Carbohydrate Analysis

The glucose and lactose were extracted using a modified method described by Ferreira Barros et al. [39]. Briefly, 5 mL of Milli-Q^®^ water was added to 1 mL of the sample and homogenized by vortexing for one minute. Afterward, the sample remained under agitation for one hour in an oven at 40 °C, and then it was centrifuged at 5000× *g* for 30 min at 4 °C. Finally, the supernatant was filtered through the 0.45 µm RC disposable syringe membrane filter (Sigma-Aldrich). The filtered samples were injected (20 µL) in triplicate into an Agilent 1260 (Agilent Technologies) HPLC system with an evaporative light scattering detector (ELSD) (gain 9, temperature evaporation and nebulization at 50 °C, pressure 3.5 bar). The determination of carbohydrates was carried out using Supelcosil 610H, 300 × 7.8 mm cation-exchange column equipped with a cation H+ microguard cartridge (Supelco), maintained at 30 °C. The mobile phase used was Milli-Q^®^ water at an isocratic flow rate of 1 mL min^−1^. Carbohydrates were identified according to their retention times compared with standard solutions. The analysis was externally calibrated using mixed standard solutions in Milli-Q^®^ water, prepared for the samples. The concentration of each sample component was determined from the area of individual peaks. The standard curve coefficients were 0.9952 and 0.9991 for glucose and lactose, respectively.

### 3.9. Statistical Analysis

The results were verified for normal distribution and homogeneity of variance. The significance of differences between means was analyzed with Tukey’s test, and the interactions between factors (storage time, addition level, and β-glucans, and the interactions between factors) were determined with ANOVA. At this stage, data were presented as means ± standard deviation. The experiment was performed in duplicate. All results were processed in Statistica 13.5 PL software (Statsoft 2017, Krakow, Poland) at a 0.05 significance level for n = 3.

## 4. Conclusions

The present study proved a significant effect of two purified, structurally-different β-glucans isolated from oats (OBG) and bacteria (curdlan) added to milk fermented by *Streptococcus thermophilus* and *Lactobacillus bulgaricus* ssp. *bulgaricus* starter cultures. After completed fermentation, a significant increase and a decrease were noted in *Streptococcus thermophilus* count in the yogurts with OBG and curdlan addition, respectively, compared to the control samples. After 28-day storage, a reduction was observed in starter cultures, significantly dependent on milk fat content and, most of all, polysaccharide structure and inclusion level. The addition of β-glucans to milk elongated its acidification process, which was longer, the lower was their concentration. The varied milk acidification dynamics caused bacterial metabolism changes, i.e., increased lactose consumption and enhanced galactose and glucose production. The experimental yogurts with curdlan had higher contents of, i.a., acetaldehyde, 2,3-pentanedione, and diacetyl, namely compounds responsible for imparting desirable sensory traits to yogurts. During 28 days of storage, due to the various functional properties of the polysaccharides, the contents of individual compounds changed significantly and were more affected by polysaccharide type than its concentration. Considering the significant effect of the oat and bacterial (curdlan) β-glucans on changes in the dynamics of the synthesis of compounds determining the organoleptic traits of yogurts, future studies should aim to determine the effects of the factors mentioned above on the sensory assessment of yogurts produced with the addition of highly-purified preparations of oat and bacterial β-glucan. It is also reasonable to determine the effect of the polysaccharides tested on the viability of probiotic cultures commonly used in the dairy industry.

## Figures and Tables

**Table 1:** Labeled standards and quantitation ions used for SIDA (stable isotope dilution assay).

Compound	Quant. Ions (*m/z*) ^a^	Labeled Standards	Ion IS (*m/z*) ^b^
Dimethyl sulfide	62	^2^H_6_ Dimethyl sulfide	68
2,3-Butanedione	86	^13^C_4_ 2,3-Butanedione	90
Acetaldehyde	44	^2^H_4_ Acetaldehyde	48
2,3-Pentanedione	100	^13^C_4_ 2,3-Butanedione	90
Acetoin	88	^13^C_4_ 2,3-Butanedione	90

^a^—Ions of analytes used for quantitation,.^b^—ions of internal standards (labeled isotopes) used for quantitation.

## Supplementary Materials

The following are available online, Table S1: Bacterial counts in yogurt with 0.25%, 0.5%, 0.75% and 1 % content of oat and bacteria (curdlan) β-glucan over 28 days of storage at 4 °C, Table S2. The lactic acid, glucose and lactose content in yogurt with 3.2% milkfat with 0.25%, 0.5%, 0.75% and 1 % content of oat and bacteria (curdlan) β-glucan during production and over 28 days of storage at 4 °C, Table S3. The lactic acid, glucose and lactose content in yogurt with 0.05% milkfat with 0.25%, 0.5%, 0.75% and 1% content of oat and bacteria (curdlan) β-glucan during production and over 28 days of storage at 4 °C, Table S4. Profile of volatile compounds in yogurt with 3.2% milkfat with 0.25%, 0.5%, 0.75% and 1% content of oat and bacteria (curdlan) β-glucan during production and over 28 days of storage at 4 °C, Table S5. Profile of volatile compounds in yogurt with 0.05% milkfat with 0.25%, 0.5%, 0.75% and 1% content of oat and bacteria (curdlan) β-glucan during production and over 28 days of storage at 4 °C, Table S6. Statistical analysis (ANOVA test) at a 0.05 significance level, Table S7. Statistical analysis (ANOVA test) at a 0.05 significance level
