# Supplementary material for: A Comprehensive Study of the Impacts of Oat β-Glucan and Bacterial Curdlan on the Activity of Commercial Starter Culture in Yogurt
Source: Molecules. 2020 Nov 19;25(22):5411. doi: 10.3390/molecules25225411 (PMC7699414; doi:10.3390/molecules25225411)
Supplement: Supplementary file 1 [file molecules-25-05411-s001.pdf]

Table S1. Bacterial counts in yogurt with 0.25 %, 0.5 %, 0.75 % and 1 % content of oat and bacteria (curdlan) β-glucan over 28 days of storage at 4°C. Values are means ± standard deviation

Factor	Addition level [%]	Time [day]	Product/ Type of β-glucan	Mean		SD	Product/ Type of β-glucan	Mean		SD	Product/ Type of β-glucan	Mean		SD
LF Str. thermophilus	0.25	0	Control	7.68	±	0.5	Oat ß-glucan	5.86	±	0.6	Curdlan	8.14	±	0.2
LF L. dulbrecki				5.15	±	0.3		4.86	±	0.1		5.00	±	0.2
FF Str. thermophilus				7.47	±	0.6		7.49	±	1.1		8.02	±	0.1
FF L. dulbrecki				4.86	±	0.2		4.89	±	0.2		5.02	±	0.2
LF Str. thermophilus		3		7.99	±	0.5		6.82	±	0.4		8.37	±	0.1
LF L. dulbrecki				5.11	±	0.2		4.82	±	0.2		4.99	±	0.2
FF Str. thermophilus				7.86	±	0.6		7.97	±	0.8		8.40	±	0.1
FF L. dulbrecki				4.90	±	0.1		4.81	±	0.2		5.01	±	0.2
LF Str. thermophilus		10		8.77	±	0.4		9.19	±	0.2		8.95	±	0.8
LF L. dulbrecki				5.05	±	0.2		4.72	±	0.1		4.98	±	0.3
FF Str. thermophilus				8.82	±	0.3		9.17	±	0.1		9.33	±	0.3
FF L. dulbrecki				5.01	±	0.2		4.63	±	0.2		4.98	±	0.3
LF Str. thermophilus		21		8.87	±	0.1		9.11	±	0.1		8.85	±	0.1
LF L. dulbrecki				4.44	±	0.1		4.23	±	0.0		4.50	±	0.1
FF Str. thermophilus				8.85	±	0.1		9.13	±	0.1		8.77	±	0.1
FF L. dulbrecki				4.59	±	0.1		4.46	±	0.2		4.51	±	0.0
LF Str. thermophilus		28		8.93	±	0.2		8.82	±	0.1		8.70	±	0.1
LF L. dulbrecki				4.51	±	0.2		4.24	±	0.1		4.47	±	0.1
FF Str. thermophilus				8.51	±	0.2		8.76	±	0.1		8.45	±	0.2
FF L. dulbrecki				4.37	±	0.0		4.18	±	0.0		4.45	±	0.1
LF Str. thermophilus	0.5	0	Control	8.54	±	0.5	Oat ß-glucan	8.66	±	0.1	Curdlan	8.69	±	0.2
LF L. dulbrecki				5.28	±	0.3		3.57	±	0.2		5.35	±	0.2
FF Str. thermophilus				8.52	±	0.1		8.33	±	0.1		9.03	±	0.1
FF L. dulbrecki				3.46	±	0.0		3.50	±	0.0		4.43	±	0.0
LF Str. thermophilus		3		8.65	±	0.4		8.73	±	0.0		8.66	±	0.1
LF L. dulbrecki				4.90	±	0.2		3.71	±	0.1		5.10	±	0.2
FF Str. thermophilus				8.49	±	0.1		8.42	±	0.1		8.64	±	0.2
FF L. dulbrecki				3.51	±	0.1		3.49	±	0.0		4.32	±	0.1
LF Str. thermophilus		10		8.90	±	0.1		8.88	±	0.1		8.56	±	0.1
LF L. dulbrecki				3.94	±	0.6		4.06	±	0.6		4.50	±	0.1
FF Str. thermophilus				8.39	±	0.0		8.62	±	0.0		7.63	±	0.0
FF L. dulbrecki				3.65	±	0.1		3.45	±	0.1		4.06	±	0.0
LF Str. thermophilus		21		9.40	±	0.3		8.73	±	0.3		8.67	±	0.1
LF L. dulbrecki				4.26	±	0.1		4.40	±	0.1		4.59	±	0.1
FF Str. thermophilus				7.61	±	0.2		7.58	±	0.1		7.67	±	0.0
FF L. dulbrecki				3.72	±	0.1		3.80	±	0.1		4.39	±	0.0
LF Str. thermophilus		28		9.27	±	0.4		8.50	±	0.4		8.70	±	0.0
LF L. dulbrecki				4.04	±	0.1		4.31	±	0.3		4.43	±	0.1
FF Str. thermophilus				8.16	±	0.2		8.32	±	0.2		7.69	±	0.2
FF L. dulbrecki				3.71	±	0.1		3.58	±	0.0		4.29	±	0.1

LF Str. thermophilus		0		8.98 ± 0.3		9.05 ± 0.1		8.72 ± 0.1
LF L. dulbrecki				4.88 ± 0.1		4.76 ± 0.0		4.70 ± 0.0
FF Str. thermophilus				8.50 ± 0.2		8.47 ± 0.2		8.65 ± 0.1
FF L. dulbrecki				4.42 ± 0.1		3.74 ± 0.1		4.23 ± 0.3
LF Str. thermophilus		3		9.07 ± 0.1		9.15 ± 0.1		8.75 ± 0.1
LF L. dulbrecki				4.80 ± 0.1		4.70 ± 0.1		4.66 ± 0.2
FF Str. thermophilus				8.48 ± 0.2		8.54 ± 0.1		8.54 ± 0.0
FF L. dulbrecki				4.25 ± 0.1		3.72 ± 0.1		4.06 ± 0.2
LF Str. thermophilus	0.75	10	Control	9.27 ± 0.3	Oat ß-glucan	9.37 ± 0.2	Curdlan	8.81 ± 0.1
LF L. dulbrecki				4.58 ± 0.1		4.54 ± 0.1		4.58 ± 0.2
FF Str. thermophilus				8.41 ± 0.0		8.69 ± 0.0		8.25 ± 0.0
FF L. dulbrecki				3.82 ± 0.1		3.67 ± 0.0		3.65 ± 0.0
LF Str. thermophilus		21		8.80 ± 0.1		8.87 ± 0.0		8.64 ± 0.2
LF L. dulbrecki				4.41 ± 0.2		4.63 ± 0.3		4.39 ± 0.3
FF Str. thermophilus				8.34 ± 0.1		8.45 ± 0.1		7.92 ± 0.6
FF L. dulbrecki				3.58 ± 0.1		3.64 ± 0.2		3.36 ± 0.1
LF Str. thermophilus		28		8.77 ± 0.1		8.86 ± 0.1		8.65 ± 0.0
LF L. dulbrecki				4.34 ± 0.4		4.38 ± 0.2		3.59 ± 0.3
FF Str. thermophilus				8.27 ± 0.2		7.60 ± 0.1		7.88 ± 0.5
FF L. dulbrecki				3.43 ± 0.2		3.62 ± 0.2		3.53 ± 0.2
LF Str. thermophilus		0		9.24 ± 0.2		9.53 ± 0.4		8.68 ± 0.1
LF L. dulbrecki				5.45 ± 0.3		4.71 ± 0.3		5.40 ± 0.1
FF Str. thermophilus				8.74 ± 0.1		8.31 ± 0.1		8.49 ± 0.1
FF L. dulbrecki				3.38 ± 0.0		3.30 ± 0.0		2.81 ± 0.0
LF Str. thermophilus		3		9.25 ± 0.2		9.44 ± 0.2		8.69 ± 0.1
LF L. dulbrecki				5.27 ± 0.2		4.66 ± 0.2		5.28 ± 0.2
FF Str. thermophilus				8.71 ± 0.1		8.36 ± 0.1		8.49 ± 0.1
FF L. dulbrecki				3.48 ± 0.0		3.35 ± 0.0		3.27 ± 0.0
LF Str. thermophilus	1	10	Control	9.24 ± 0.2	Oat ß-glucan	9.20 ± 0.1	Curdlan	8.67 ± 0.0
LF L. dulbrecki				4.84 ± 0.1		4.54 ± 0.1		5.01 ± 0.1
FF Str. thermophilus				8.60 ± 0.0		8.47 ± 0.1		8.47 ± 0.0
FF L. dulbrecki				3.73 ± 0.1		3.49 ± 0.1		4.42 ± 0.1
LF Str. thermophilus		21		8.30 ± 0.1		8.89 ± 0.1		8.63 ± 0.0
LF L. dulbrecki				4.71 ± 0.1		4.43 ± 0.0		4.77 ± 0.0
FF Str. thermophilus				8.60 ± 0.1		8.43 ± 0.0		8.43 ± 0.0
FF L. dulbrecki				3.46 ± 0.0		3.30 ± 0.0		4.36 ± 0.0
LF Str. thermophilus		28		6.43 ± 0.1		8.67 ± 0.1		8.55 ± 0.1
LF L. dulbrecki				4.37 ± 0.1		4.18 ± 0.1		4.48 ± 0.1
FF Str. thermophilus				4.47 ± 0.0		7.55 ± 0.0		7.65 ± 0.0
FF L. dulbrecki				3.01 ± 0.0		3.22 ± 0.0		4.29 ± 0.0

LF – low fat (0.05 % milkfat)
FF – full fat (3.2 % milkfat)

Table S2. The lactic acid, glucose and lactose content in yogurt with 3.2 % milkfat with 0.25 %, 0.5 %, 0.75 % and 1 % content of oat and bacteria (curdlan) β-glucan during production and over 28 days of storage at 4°C. Values are means ± standard deviation.

Compound (mg/kg)	Addition level [%]	Product	Time	Mean		SD	Time [h]	Mean		SD	Time [days]	Mean		SD	Time [days]	Mean		SD	Time [days]	Mean		SD	Time [days]	Mean		SD
lactic acid	0.00	Oat ß-glucan	0	0.05	±	0.0	4	0.26	±	0.0	3	0.37	±	0.0	10	0.39	±	0.0	21	0.40	±	0.0	28	0.41	±	0.0
lactose				49.94	±	0.9		48.22	±	0.7		46.77	±	0.2		46.42	±	0.6		46.57	±	0.5		46.67	±	0.4
glucose				0.08	±	0.0		0.08	±	0.0		0.08	±	0.0		0.08	±	0.0		0.09	±	0.0		0.09	±	0.0
lactic acid		Curdlan		0.05	±	0.0		0.26	±	0.0		0.37	±	0.0		0.39	±	0.0		0.40	±	0.0		0.41	±	0.0
lactose				49.94	±	0.9		48.22	±	0.7		46.77	±	0.2		46.42	±	0.6		46.57	±	0.5		46.67	±	0.4
glucose				0.08	±	0.0		0.08	±	0.0		0.08	±	0.0		0.08	±	0.0		0.09	±	0.0		0.09	±	0.0
lactic acid	0.25	Oat ß-glucan		0.05	±	0.0		0.22	±	0.0		0.35	±	0.0		0.37	±	0.0		0.38	±	0.0		0.39	±	0.0
lactose				49.85	±	0.8		48.45	±	0.7		47.55	±	0.3		46.65	±	0.3		46.35	±	0.2		46.89	±	0.4
glucose				0.10	±	0.0		0.08	±	0.0		0.06	±	0.0		0.06	±	0.0		0.07	±	0.0		0.07	±	0.0
lactic acid		Curdlan		0.05	±	0.0		0.21	±	0.0		0.31	±	0.0		0.32	±	0.0		0.38	±	0.0		0.39	±	0.0
lactose				49.09	±	0.5		48.15	±	0.4		47.31	±	0.2		46.90	±	0.3		47.08	±	0.8		46.67	±	0.9
glucose				0.06	±	0.0		0.05	±	0.0		0.04	±	0.0		0.04	±	0.0		0.04	±	0.0		0.04	±	0.0
lactic acid	0.50	Oat ß-glucan		0.05	±	0.0		0.22	±	0.0		0.35	±	0.0		0.37	±	0.0		0.37	±	0.0		0.39	±	0.0
lactose				49.57	±	0.8		48.25	±	0.7		47.31	±	0.3		46.42	±	0.3		46.18	±	0.2		46.64	±	0.4
glucose				0.05	±	0.0		0.04	±	0.0		0.02	±	0.0		0.03	±	0.0		0.03	±	0.0		0.03	±	0.0
lactic acid		Curdlan		0.05	±	0.0		0.23	±	0.0		0.37	±	0.0		0.37	±	0.0		0.38	±	0.0		0.39	±	0.0
lactose				47.91	±	0.4		46.83	±	0.4		45.72	±	0.2		45.37	±	0.3		45.87	±	0.8		45.54	±	0.9
glucose				0.03	±	0.0		0.02	±	0.0		0.02	±	0.0		0.02	±	0.0		0.02	±	0.0		0.02	±	0.0
lactic acid	0.75	Oat ß-glucan		0.05	±	0.0		0.20	±	0.0		0.35	±	0.0		0.38	±	0.0		0.39	±	0.0		0.40	±	0.0
lactose				48.95	±	0.8		47.82	±	0.7		46.69	±	0.3		45.79	±	0.3		45.49	±	0.2		46.00	±	0.3
glucose				0.24	±	0.0		0.20	±	0.0		0.16	±	0.0		0.18	±	0.0		0.19	±	0.0		0.19	±	0.0
lactic acid		Curdlan		0.05	±	0.0		0.21	±	0.0		0.36	±	0.0		0.38	±	0.0		0.40	±	0.0		0.40	±	0.0
lactose				48.41	±	0.4		47.48	±	0.4		46.27	±	0.2		45.77	±	0.3		46.21	±	0.8		45.93	±	0.9
glucose				0.14	±	0.0		0.13	±	0.0		0.13	±	0.0		0.12	±	0.0		0.11	±	0.0		0.11	±	0.0
lactic acid	1.00	Oat ß-glucan		0.05	±	0.0		0.35	±	0.0		0.34	±	0.0		0.36	±	0.0		0.37	±	0.0		0.37	±	0.0
lactose				48.56	±	0.8		46.54	±	0.6		46.49	±	0.3		45.64	±	0.3		45.33	±	0.2		45.86	±	0.3
glucose				0.36	±	0.0		0.30	±	0.0		0.25	±	0.0		0.27	±	0.0		0.29	±	0.0		0.32	±	0.0
lactic acid		Curdlan		0.05	±	0.0		0.23	±	0.0		0.33	±	0.0		0.35	±	0.0		0.37	±	0.0		0.38	±	0.0
lactose				48.04	±	0.4		47.02	±	0.4		46.22	±	0.2		45.71	±	0.3		46.12	±	0.8		45.80	±	0.9
glucose				0.20	±	0.0		0.20	±	0.0		0.19	±	0.0		0.18	±	0.0		0.20	±	0.0		0.20	±	0.0

Table S3. The lactic acid, glucose and lactose content in yogurt with 0.05 % milkfat with 0.25 %, 0.5 %, 0.75 % and 1 % content of oat and bacteria (curdlan) β-glucan during production and over 28 days of storage at 4°C. Values are means ± standard deviation.

Compound (mg/kg)	Addition level [%]	Product	Time	Mean		SD	Time [h]	Mean		SD	Time [days]	Mean		SD	Time [days]	Mean		SD	Time [days]	Mean		SD	Time [days]	Mean		SD
lactic acid	0.00	Oat ß-glucan	0	0.05	±	0.0	4	0.32	±	0.0	3	0.34	±	0.0	10	0.37	±	0.0	21	0.40	±	0.0	28	0.40	±	0.0
lactose				48.54	±	0.2		46.48	±	0.6		45.70	±	0.2		45.05	±	0.3		44.61	±	0.3		44.79	±	0.2
glucose				0.04	±	0.0		0.04	±	0.0		0.03	±	0.0		0.04	±	0.0		0.04	±	0.0		0.04	±	0.0
lactic acid		Curdlan		0.05	±	0.0		0.32	±	0.0		0.34	±	0.0		0.37	±	0.0		0.40	±	0.0		0.40	±	0.0
lactose				48.54	±	0.2		46.48	±	0.6		45.70	±	0.2		45.05	±	0.3		44.61	±	0.3		44.79	±	0.2
glucose				0.04	±	0.0		0.04	±	0.0		0.03	±	0.0		0.04	±	0.0		0.04	±	0.0		0.04	±	0.0
lactic acid	0.25	Oat ß-glucan		0.05	±	0.0		0.22	±	0.0		0.32	±	0.0		0.36	±	0.0		0.38	±	0.0		0.38	±	0.0
lactose				48.50	±	0.8		47.12	±	0.7		45.47	±	0.3		43.81	±	0.3		43.52	±	0.2		44.01	±	0.3
glucose				0.17	±	0.0		0.15	±	0.0		0.13	±	0.0		0.14	±	0.0		0.15	±	0.0		0.15	±	0.0
lactic acid		Curdlan		0.05	±	0.0		0.21	±	0.0		0.29	±	0.0		0.33	±	0.0		0.36	±	0.0		0.35	±	0.0
lactose				47.58	±	0.4		45.99	±	0.4		44.52	±	0.2		43.39	±	0.3		43.73	±	0.8		43.51	±	0.8
glucose				0.08	±	0.0		0.08	±	0.0		0.11	±	0.0		0.10	±	0.0		0.10	±	0.0		0.10	±	0.0
lactic acid	0.50	Oat ß-glucan		0.05	±	0.0		0.21	±	0.0		0.30	±	0.0		0.34	±	0.0		0.35	±	0.0		0.35	±	0.0
lactose				48.50	±	0.8		47.12	±	0.7		45.47	±	0.3		43.81	±	0.3		43.53	±	0.2		44.10	±	0.7
glucose				0.17	±	0.0		0.15	±	0.0		0.13	±	0.0		0.14	±	0.0		0.15	±	0.0		0.15	±	0.0
lactic acid		Curdlan		0.13	±	0.0		0.37	±	0.0		0.37	±	0.0		0.36	±	0.0		0.36	±	0.0		0.36	±	0.0
lactose				48.02	±	0.4		46.67	±	0.6		45.55	±	0.4		45.42	±	0.6		44.96	±	0.8		44.60	±	0.8
glucose				0.08	±	0.0		0.08	±	0.0		0.07	±	0.0		0.06	±	0.0		0.06	±	0.0		0.05	±	0.0
lactic acid	0.75	Oat ß-glucan		0.05	±	0.0		0.05	±	0.0		0.08	±	0.0		0.26	±	0.0		0.36	±	0.0		0.38	±	0.0
lactose				48.50	±	0.8		47.12	±	0.7		45.47	±	0.3		43.81	±	0.3		42.96	±	0.2		43.33	±	0.3
glucose				0.17	±	0.0		0.15	±	0.0		0.13	±	0.0		0.14	±	0.0		0.15	±	0.0		0.16	±	0.0
lactic acid		Curdlan		0.05	±	0.0		0.28	±	0.0		0.31	±	0.0		0.36	±	0.0		0.39	±	0.0		0.40	±	0.0
lactose				47.58	±	0.4		46.03	±	0.4		45.39	±	0.4		43.57	±	0.9		43.13	±	0.4		43.55	±	0.5
glucose				0.08	±	0.0		0.08	±	0.0		0.07	±	0.0		0.06	±	0.0		0.06	±	0.0		0.05	±	0.0
lactic acid	1.00	Oat ß-glucan		0.05	±	0.0		0.21	±	0.0		0.23	±	0.0		0.34	±	0.0		0.37	±	0.0		0.37	±	0.0
lactose				48.07	±	0.7		47.17	±	1.0		47.73	±	0.7		46.19	±	0.7		46.15	±	0.8		46.03	±	0.2
glucose				0.17	±	0.0		0.15	±	0.0		0.13	±	0.0		0.14	±	0.0		0.15	±	0.0		0.16	±	0.0
lactic acid		Curdlan		0.04	±	0.0		0.12	±	0.0		0.22	±	0.0		0.43	±	0.0		0.45	±	0.0		0.46	±	0.0
lactose				47.90	±	0.6		47.48	±	0.5		46.62	±	0.2		44.91	±	0.3		45.00	±	0.8		44.62	±	0.8
glucose				0.08	±	0.0		0.07	±	0.0		0.07	±	0.0		0.06	±	0.0		0.06	±	0.0		0.05	±	0.0

Table S4. Profile of volatile compounds in yogurt with 3.2% milkfat with 0.25%, 0.5%, 0.75% and 1% content of oat and bacteria (curdlan) β-glucan during production and over 28 days of storage at 4°C. Values are means ± standard deviation.

Compound (µg/kg)	Addition level [%]	Product	Time	Mean		SD	Time [h]	Mean		SD	Time [days]	Mean		SD	Time [days]	Mean		SD	Time [days]	Mean		SD	Time [days]	Mean		SD
dimethyl sulfide	0.00	Oat ß-glucan	0	115.69	±	3	4	120.22	±	9	3	168.21	±	3	10	159.43	±	7	21	140.67	±	3	28	125.13	±	8
2,3-butanedione				1090.27	±	14		1640.60	±	150		2269.80	±	107		3107.78	±	58		3472.90	±	72		4116.52	±	105
acetaldehyde				82.50	±	1		633.88	±	16		4019.14	±	62		3524.35	±	175		2590.94	±	163		2446.99	±	22
acetoin				221.97	±	4		4867.83	±	177		2587.43	±	136		2658.12	±	109		2932.54	±	54		4330.07	±	194
2,3-pentanedione				882.64	±	6		1729.37	±	34		2026.75	±	26		1805.27	±	62		2580.26	±	70		3056.55	±	87
acetic acid				114.05	±	1		3645.18	±	45		5682.18	±	25		6084.76	±	110		6350.19	±	496		8103.37	±	301
dimethyl sulfide		Curdlan		115.69	±	3		120.22	±	9		168.21	±	3		159.43	±	7		140.67	±	3		125.13	±	8
2,3-butanedione				1090.27	±	14		1640.60	±	150		2269.80	±	107		3107.78	±	58		3472.90	±	72		4116.52	±	105
acetaldehyde				82.50	±	1		633.88	±	16		4019.14	±	62		3524.35	±	175		2590.94	±	163		2446.99	±	22
acetoin				221.97	±	4		4867.83	±	177		2587.43	±	136		2658.12	±	109		2932.54	±	54		4330.07	±	194
2,3-pentanedione				882.64	±	6		1729.37	±	34		2026.75	±	26		1805.27	±	62		2580.26	±	70		3056.55	±	87
acetic acid				114.05	±	1		3645.18	±	45		5682.18	±	25		6084.76	±	110		6350.19	±	496		8103.37	±	301
dimethyl sulfide	0.25	Oat ß-glucan		133.08	±	1		89.86	±	1		109.80	±	2		100.88	±	4		102.50	±	2		97.80	±	1
2,3-butanedione				1142.50	±	51		1158.00	±	70		1168.50	±	48		1328.50	±	24		1530.25	±	32		3355.25	±	47
acetaldehyde				179.30	±	6		265.36	±	1		3948.23	±	308		2297.66	±	69		1346.94	±	41		1199.52	±	83
acetoin				2370.14	±	26		3274.22	±	28		3022.21	±	224		2525.16	±	215		2362.53	±	107		2493.43	±	64
2,3-pentanedione				2870.50	±	132		3182.00	±	32		4939.75	±	69		5150.50	±	60		6090.50	±	21		2739.00	±	137
acetic acid				0.00	±	0		264.50	±	2		1006.41	±	89		1962.50	±	106		3602.88	±	326		4083.09	±	273
dimethyl sulfide		Curdlan		185.60	±	4		218.35	±	4		188.21	±	13		183.57	±	7		146.87	±	4		92.01	±	6
2,3-butanedione				1338.50	±	46		1295.50	±	10		1502.25	±	77		1598.25	±	11		1853.25	±	52		2029.50	±	62
acetaldehyde				313.76	±	15		701.39	±	59		4609.48	±	401		3216.27	±	147		3014.57	±	181		2763.15	±	124
acetoin				1738.31	±	4		2076.27	±	103		2276.93	±	134		3281.81	±	267		5272.27	±	225		4802.67	±	253
2,3-pentanedione				6499.50	±	48		3363.00	±	44		6124.00	±	57		5205.50	±	21		5086.25	±	30		2581.00	±	213
acetic acid				106.31	±	3		3951.68	±	18		4825.17	±	216		5118.26	±	288		5117.73	±	195		5080.10	±	237
dimethyl sulfide	0.50	Oat ß-glucan		130.06	±	10		91.23	±	4		115.85	±	9		101.45	±	7		99.30	±	3		103.07	±	8
2,3-butanedione				1184.65	±	11		1143.80	±	17		1134.06	±	62		1283.00	±	74		1492.04	±	105		3378.12	±	189
acetaldehyde				147.82	±	10		185.75	±	5		3148.39	±	298		1816.52	±	120		1017.21	±	33		986.32	±	79
acetoin				2647.80	±	110		2977.40	±	16		3410.87	±	129		2704.25	±	117		2586.90	±	120		2695.16	±	116

2,3-pentanedione		Curdlan	3367.00 ± 128	3502.00 ± 70	5384.75 ± 85	4747.25 ± 158	4954.00 ± 68	4476.50 ± 126
acetic acid			0.00 ± 0	325.88 ± 9	882.82 ± 78	1721.49 ± 93	3110.42 ± 218	3581.66 ± 239
dimethyl sulfide			194.75 ± 13	188.06 ± 10	178.68 ± 4	180.14 ± 6	144.52 ± 5	88.18 ± 9
2,3-butanedione			1368.24 ± 52	1413.57 ± 45	1461.18 ± 186	1542.52 ± 56	1757.26 ± 71	2042.61 ± 122
acetaldehyde			233.38 ± 10	495.80 ± 11	3409.73 ± 125	2288.27 ± 54	2131.29 ± 66	1910.21 ± 120
acetoin			2069.06 ± 118	2214.03 ± 109	2594.86 ± 41	3514.57 ± 136	5886.37 ± 397	5561.59 ± 223
2,3-pentanedione			4052.50 ± 78	2800.50 ± 64	4987.25 ± 62	3296.75 ± 98	4009.25 ± 13	3943.25 ± 103
acetic acid			1843.13 ± 108	3499.96 ± 95	4379.28 ± 256	4486.68 ± 158	4613.16 ± 463	4557.16 ± 227
dimethyl sulfide	0.75	Oat ß-glucan	104.72 ± 1	79.07 ± 4	91.44 ± 5	87.92 ± 6	86.06 ± 3	89.33 ± 7
2,3-butanedione			1070.10 ± 56	1074.32 ± 16	3319.03 ± 123	3938.16 ± 160	4116.83 ± 321	4480.81 ± 219
acetaldehyde			159.00 ± 1	190.50 ± 6	3571.00 ± 128	2071.00 ± 66	1182.25 ± 59	1078.25 ± 44
acetoin			2222.00 ± 9	2524.00 ± 45	3018.25 ± 34	2326.75 ± 34	2261.50 ± 132	2360.00 ± 45
2,3-pentanedione			1005.00 ± 8	1034.00 ± 13	1084.25 ± 30	1126.00 ± 41	3076.75 ± 42	5030.50 ± 83
acetic acid			0.00 ± 0	941.00 ± 53	1395.25 ± 68	3317.25 ± 199	4122.00 ± 96	5461.75 ± 96
dimethyl sulfide		Curdlan	159.71 ± 1	164.70 ± 13	160.14 ± 4	156.11 ± 5	128.58 ± 11	81.42 ± 12
2,3-butanedione			2727.31 ± 62	2058.96 ± 82	5182.95 ± 133	6987.71 ± 299	6609.07 ± 424	5711.34 ± 312
acetaldehyde			247.50 ± 3	552.00 ± 13	3869.00 ± 70	2553.50 ± 81	2383.25 ± 107	2795.56 ± 121
acetoin			1654.00 ± 5	1857.50 ± 6	2221.50 ± 28	3024.25 ± 32	5013.75 ± 27	4591.25 ± 123
2,3-pentanedione			1835.00 ± 62	2552.00 ± 13	1021.25 ± 23	1066.25 ± 40	3540.00 ± 31	5483.25 ± 84
acetic acid			0.00 ± 0	984.00 ± 10	1077.75 ± 58	1913.25 ± 68	2036.25 ± 77	4555.75 ± 112
dimethyl sulfide	1.00	Oat ß-glucan	118.50 ± 1	84.00 ± 1	104.25 ± 5	98.00 ± 3	97.75 ± 2	95.50 ± 2
2,3-butanedione			1005.00 ± 20	1045.00 ± 64	3288.75 ± 165	3920.00 ± 39	4054.50 ± 59	4277.00 ± 45
acetaldehyde			162.77 ± 11	193.52 ± 18	3466.76 ± 328	2000.21 ± 132	1120.07 ± 36	1086.07 ± 87
acetoin			2275.04 ± 154	2557.99 ± 111	2929.20 ± 247	2245.91 ± 97	2142.57 ± 96	2377.00 ± 169
2,3-pentanedione			967.24 ± 6	1089.71 ± 18	1040.51 ± 74	1074.93 ± 30	2886.89 ± 54	5012.43 ± 299
acetic acid			0.00 ± 0	1198.71 ± 66	1459.39 ± 152	2947.35 ± 505	4553.33 ± 71	5852.93 ± 155
dimethyl sulfide		Curdlan	183.50 ± 4	179.00 ± 6	177.75 ± 2	174.25 ± 5	142.00 ± 7	86.75 ± 8
2,3-butanedione			2565.50 ± 126	1999.00 ± 12	5132.75 ± 90	6955.00 ± 49	6513.50 ± 60	5451.75 ± 157
acetaldehyde			256.98 ± 11	545.94 ± 12	3754.54 ± 138	2464.62 ± 122	2319.29 ± 81	2473.61 ± 290
acetoin			1637.29 ± 79	1820.46 ± 48	2084.67 ± 68	2919.06 ± 117	4766.20 ± 109	4352.69 ± 145
2,3-pentanedione			1795.65 ± 21	2475.65 ± 69	948.16 ± 16	1019.90 ± 81	3331.21 ± 96	5147.36 ± 208
acetic acid			0.00 ± 0	987.64 ± 1	990.76 ± 8	1063.87 ± 55	1297.98 ± 95	3827.26 ± 157

Table S5. Profile of volatile compounds in yogurt with 0.05% milkfat with 0.25%, 0.5%, 0.75% and 1% content of oat and bacteria (curdlan) β-glucan during production and over 28 days of storage at 4°C. Values are means ± standard deviation.

Compound (μg/kg)	Addition level [%]	Product	Time	Mean		SD	Time [h]	Mean		SD	Time [days]	Mean		SD	Time [days]	Mean		SD	Time [days]	Mean		SD	Time [days]	Mean		SD
dimethyl sulfide	0.00	Oat ß-glucan	0	126.91	±	6	4	112.35	±	4	3	157.56	±	4	10	162.30	±	4	21	176.05	±	4	28	157.43	±	3
2,3-butanedione	0.00	Oat ß-glucan	0	613.90	±	11	4	828.16	±	9	3	1078.44	±	20	10	1122.70	±	17	21	1239.02	±	11	28	1265.77	±	16
acetaldehyde	0.00	Oat ß-glucan	0	2118.93	±	17	4	5084.83	±	203	3	5890.91	±	208	10	6770.86	±	148	21	6918.07	±	107	28	7203.47	±	231
acetoin	0.00	Oat ß-glucan	0	2336.59	±	54	4	4933.37	±	115	3	4179.77	±	138	10	3728.52	±	242	21	3353.99	±	58	28	3239.82	±	10
2,3-pentanedione	0.00	Oat ß-glucan	0	648.40	±	26	4	1339.15	±	61	3	2231.51	±	70	10	2425.17	±	76	21	2585.63	±	38	28	2163.61	±	74
acetic acid	0.00	Oat ß-glucan	0	346.05	±	6	4	4044.83	±	191	3	7954.00	±	57	10	8004.67	±	62	21	8510.75	±	411	28	9370.87	±	254
dimethyl sulfide	0.00	Curdlan	0	126.91	±	6	4	112.35	±	4	3	157.56	±	4	10	162.30	±	4	21	176.05	±	4	28	157.43	±	3
2,3-butanedione	0.00	Curdlan	0	613.90	±	11	4	828.16	±	9	3	1078.44	±	20	10	1122.70	±	17	21	1239.02	±	11	28	1265.77	±	16
acetaldehyde	0.00	Curdlan	0	2118.93	±	17	4	5084.83	±	203	3	5890.91	±	208	10	6770.86	±	148	21	6918.07	±	107	28	7203.47	±	231
acetoin	0.00	Curdlan	0	2336.59	±	54	4	4933.37	±	115	3	4179.77	±	138	10	3728.52	±	242	21	3353.99	±	58	28	3239.82	±	10
2,3-pentanedione	0.00	Curdlan	0	648.40	±	26	4	1339.15	±	61	3	2231.51	±	70	10	2425.17	±	76	21	2585.63	±	38	28	2163.61	±	74
acetic acid	0.00	Curdlan	0	346.05	±	6	4	4044.83	±	191	3	7954.00	±	57	10	8004.67	±	62	21	8510.75	±	411	28	9370.87	±	254
dimethyl sulfide	0.25	Oat ß-glucan	0	93.00	±	1	4	278.00	±	13	3	380.00	±	5	10	443.50	±	4	21	491.25	±	14	28	317.75	±	15
2,3-butanedione	0.25	Oat ß-glucan	0	98.50	±	1	4	103.50	±	1	3	237.25	±	7	10	593.75	±	6	21	627.00	±	5	28	665.25	±	29
acetaldehyde	0.25	Oat ß-glucan	0	352.00	±	7	4	543.50	±	24	3	1965.50	±	91	10	2052.75	±	94	21	2163.50	±	80	28	2351.00	±	64
acetoin	0.25	Oat ß-glucan	0	1034.00	±	13	4	2310.50	±	92	3	3175.25	±	230	10	6590.75	±	59	21	4241.00	±	21	28	3954.25	±	41
2,3-pentanedione	0.25	Oat ß-glucan	0	663.30	±	5	4	682.44	±	8	3	715.61	±	20	10	743.16	±	27	21	2030.66	±	28	28	3320.13	±	54
acetic acid	0.25	Oat ß-glucan	0	0.00	±	0	4	273.37	±	8	3	701.06	±	38	10	1623.09	±	134	21	3144.65	±	123	28	3460.00	±	103
dimethyl sulfide	0.25	Curdlan	0	36.50	±	2	4	113.50	±	5	3	102.00	±	5	10	254.75	±	7	21	446.75	±	22	28	63.50	±	6
2,3-butanedione	0.25	Curdlan	0	200.50	±	2	4	239.50	±	10	3	641.50	±	14	10	931.50	±	16	21	1056.25	±	17	28	1164.75	±	40
acetaldehyde	0.25	Curdlan	0	972.00	±	17	4	943.50	±	54	3	2494.75	±	46	10	1978.25	±	33	21	1292.25	±	9	28	575.00	±	27
acetoin	0.25	Curdlan	0	653.50	±	1	4	981.50	±	4	3	6451.00	±	135	10	6814.75	±	127	21	7222.25	±	28	28	2316.50	±	29
2,3-pentanedione	0.25	Curdlan	0	1082.12	±	60	4	1684.47	±	47	3	645.14	±	11	10	693.95	±	55	21	2203.45	±	119	28	3562.03	±	27
acetic acid	0.25	Curdlan	0	784.74	±	65	4	2512.27	±	205	3	4042.26	±	241	10	4073.74	±	105	21	4224.05	±	318	28	4092.82	±	229
dimethyl sulfide	0.50	Oat ß-glucan	0	13.27	±	1	4	141.51	±	12	3	211.58	±	24	10	124.46	±	4	21	172.22	±	7	28	189.83	±	11
2,3-butanedione	0.50	Oat ß-glucan	0	83.92	±	7	4	104.74	±	7	3	173.35	±	13	10	269.45	±	11	21	478.71	±	18	28	483.20	±	26
acetaldehyde	0.50	Oat ß-glucan	0	1993.29	±	19	4	2033.60	±	1	3	1986.52	±	70	10	2041.26	±	133	21	2098.67	±	73	28	2131.86	±	75
acetoin	0.50	Oat ß-glucan	0	3655.51	±	205	4	6904.74	±	355	3	6868.23	±	464	10	5676.93	±	619	21	4870.41	±	169	28	4115.58	±	164

2,3-pentanedione			1500.18 ± 39	1741.48 ± 120	2422.18 ± 201	2728.63 ± 380	3053.88 ± 212	1605.31 ± 207
acetic acid			0.00 ± 0	206.57 ± 12	821.02 ± 73	1600.99 ± 86	2939.19 ± 266	3330.94 ± 223
dimethyl sulfide		Curdlan	359.22 ± 21	318.09 ± 19	208.94 ± 11	97.28 ± 5	101.53 ± 8	122.55 ± 4
2,3-butanedione			647.43 ± 40	1009.75 ± 15	1049.18 ± 71	1372.66 ± 57	1410.64 ± 103	1449.38 ± 81
acetaldehyde			1718.70 ± 71	5449.55 ± 9	5480.42 ± 136	5711.64 ± 228	5264.34 ± 338	5147.48 ± 263
acetoin			1259.20 ± 124	2309.03 ± 144	2000.93 ± 84	1992.33 ± 108	1756.62 ± 131	1803.03 ± 135
2,3-pentanedione			1290.18 ± 20	1776.70 ± 58	2923.61 ± 190	2754.86 ± 351	2556.49 ± 165	1322.17 ± 117
acetic acid			714.11 ± 15	2254.96 ± 89	4072.73 ± 238	4172.61 ± 147	4290.24 ± 431	4400.90 ± 294
dimethyl sulfide	0.75	Oat ß-glucan	15.50 ± 1	17.50 ± 1	27.25 ± 2	158.25 ± 11	222.50 ± 6	231.00 ± 2
2,3-butanedione			84.50 ± 1	106.50 ± 1	184.50 ± 9	288.00 ± 5	520.75 ± 9	495.25 ± 7
acetaldehyde			2430.00 ± 17	2141.50 ± 50	2183.75 ± 39	2181.00 ± 94	2277.25 ± 53	2319.25 ± 44
acetoin			3572.50 ± 56	6810.50 ± 68	6814.00 ± 241	6242.25 ± 53	5228.50 ± 45	4784.25 ± 171
2,3-pentanedione			219.70 ± 16	1842.70 ± 74	4409.80 ± 227	7026.02 ± 400	6804.51 ± 128	2212.88 ± 150
acetic acid			0.00 ± 0	219.50 ± 6	891.25 ± 12	1748.00 ± 72	3263.50 ± 234	3486.75 ± 120
dimethyl sulfide		Curdlan	430.00 ± 6	393.50 ± 5	264.00 ± 17	123.50 ± 6	127.50 ± 3	149.25 ± 4
2,3-butanedione			652.50 ± 6	1053.50 ± 33	1115.25 ± 66	1467.00 ± 18	1492.00 ± 14	1485.00 ± 19
acetaldehyde			269.45 ± 7	5738.50 ± 123	6025.25 ± 16	6104.25 ± 39	5709.25 ± 264	5599.75 ± 224
acetoin			1210.00 ± 53	2331.50 ± 39	2061.75 ± 47	2063.25 ± 33	1801.25 ± 46	1789.25 ± 33
2,3-pentanedione			465.13 ± 5	2099.04 ± 42	3104.40 ± 91	3308.99 ± 123	4029.69 ± 65	1805.41 ± 128
acetic acid			1765.00 ± 23	3467.50 ± 64	4422.50 ± 218	4556.75 ± 107	4630.50 ± 227	4606.25 ± 187
dimethyl sulfide	1.00	Oat ß-glucan	148.70 ± 1	101.06 ± 0	108.38 ± 7	104.49 ± 5	98.30 ± 5	96.41 ± 4
2,3-butanedione			837.50 ± 6	988.00 ± 12	1003.75 ± 5	1054.00 ± 35	1041.75 ± 23	1092.25 ± 23
acetaldehyde			1813.89 ± 14	1961.06 ± 29	1899.04 ± 161	2057.99 ± 322	1917.64 ± 71	2110.11 ± 152
acetoin			2577.10 ± 155	3339.77 ± 28	4075.93 ± 220	4472.43 ± 250	5401.89 ± 290	6704.09 ± 623
2,3-pentanedione			1885.10 ± 49	2075.79 ± 148	3079.27 ± 216	3197.28 ± 171	3712.21 ± 92	1775.38 ± 176
acetic acid			0.00 ± 0	278.50 ± 2	742.59 ± 29	1497.70 ± 81	2756.14 ± 238	2935.26 ± 138
dimethyl sulfide		Curdlan	221.28 ± 4	234.92 ± 6	301.77 ± 8	398.85 ± 17	392.52 ± 9	382.00 ± 21
2,3-butanedione			1836.00 ± 44	2248.50 ± 57	2950.25 ± 132	3148.00 ± 174	2469.25 ± 165	2111.75 ± 106
acetaldehyde			1612.07 ± 112	5020.54 ± 35	5589.98 ± 436	5241.20 ± 213	4752.90 ± 251	4726.32 ± 223
acetoin			1028.94 ± 46	1894.99 ± 1	1695.56 ± 101	1736.11 ± 117	1507.16 ± 113	1544.07 ± 136
2,3-pentanedione			4134.59 ± 159	2119.17 ± 77	3693.49 ± 71	3230.32 ± 137	3108.15 ± 63	1573.05 ± 136
acetic acid			1603.52 ± 94	3119.65 ± 134	3799.87 ± 168	3906.04 ± 220	3895.06 ± 143	4103.90 ± 150

Table S6. Statistical analysis (ANOVA test) at a 0.05 significance level

Factor	dimethyl sulfide	2,3-butanedione	acetaldehyde	acetoin	2,3-pentanedione	acetic acid	lactic acid	lactose	glucose
Addition level [A]	<0.001	<0.001	<0.01	<0.01	<0.001	<0.01	<0.01	<0.001	<0.01
Fat content [F]	<0.001	<0.001	<0.01	<0.01	<0.001	<0.01	<0.01	<0.001	<0.01
Type of β-glucan [BG]	<0.001	<0.001	<0.01	<0.01	0.001	<0.01	<0.01	<0.001	<0.01
Time [T]	<0.001	<0.001	<0.01	<0.01	<0.001	<0.01	<0.01	<0.001	<0.01
[A]×[F]	<0.001	<0.001	<0.01	<0.01	<0.001	<0.01	<0.01	<0.001	<0.01
[A]×[BG]	<0.001	<0.001	<0.01	<0.01	<0.001	<0.01	<0.01	0.079	<0.01
[F]×[BG]	0.044	0.230	<0.01	<0.01	<0.001	<0.01	<0.01	0.972	<0.01
[A]×[T]	<0.001	<0.001	<0.01	<0.01	<0.001	<0.01	<0.01	<0.001	<0.01
[F]×[T]	<0.001	<0.001	<0.01	<0.01	<0.001	<0.01	<0.01	<0.001	<0.01
[BG]×[T]	<0.001	<0.001	<0.01	<0.01	<0.001	<0.01	<0.01	<0.001	<0.01
[A]×[F]×[BG]	<0.001	<0.001	<0.01	<0.01	<0.001	<0.01	<0.01	<0.001	<0.01
[A]×[F]×[T]	<0.001	<0.001	<0.01	<0.01	<0.001	<0.01	<0.01	<0.001	<0.01
[A]×[BG]×[T]	<0.001	<0.001	<0.01	<0.01	<0.001	<0.01	<0.01	0.003	<0.01
[F]×[BG]×[T]	<0.001	<0.001	<0.01	<0.01	<0.001	<0.01	<0.01	0.762	<0.01
[A]×[F]×[BG]×[T]	<0.001	<0.001	<0.01	<0.01	<0.001	<0.01	<0.01	0.418	<0.01

Table S7. Statistical analysis (ANOVA test) at a 0.05 significance level

	LF *Str. thermophilus*	LF *L. dulbrecki*	FF *Str. thermophilus*	FF *L. dulbrecki*
Addition level [A]	<0.001	<0.01	<0.01	<0.01
Fat content [F]	0.030	<0.01	<0.01	<0.01
Time [T]	<0.001	<0.01	<0.01	<0.01
[A]×[BG]	<0.001	<0.01	<0.01	<0.01
[A]×[T]	<0.001	<0.01	<0.01	<0.01
[BG]×[T]	<0.001	<0.01	<0.01	<0.01
[A]×[BG]×[T]	<0.001	<0.01	<0.01	<0.01

LF – low fat (0.05 % milkfat)

FF – full fat (3.2 % milkfat)
